# An ecological analysis of PM_2.5_ concentrations and lung cancer mortality rates in China

**DOI:** 10.1136/bmjopen-2015-009452

**Published:** 2015-11-24

**Authors:** Jingying Fu, Dong Jiang, Gang Lin, Kun Liu, Qiao Wang

**Affiliations:** 1 State Key Laboratory of Resources and Environmental Information System, Institute of Geographical Sciences and Natural Resources Research, Chinese Academy of Sciences, Beijing, China; 2 College of Geoscience and Surveying Engineering, China University of Mining & Technology (Beijing), Beijing, China; 3 China Aero Geophysical Survey & Remote Sensing Center for Land and Resources, Beijing, China; 4 Institute of Geographical Sciences and Nature Resources Research, Chinese Academy of Sciences, Beijing, China; 5 University of Chinese Academy of Sciences, Beijing, China; 6 Satellite Environmental Application Center, Ministry of Environmental Protection, Beijing, China

**Keywords:** PM2.5, population, lung cancer mortality, GWR, ecologic study

## Abstract

**Objective:**

To explore the association between Particulate Matter (PM)_2.5_ (particles with an aerodynamic diameter less than 2.5 µm) and lung cancer mortality rates and to estimate the potential risk of lung cancer mortality related to exposure to high PM_2.5_ concentrations.

**Design:**

Geographically weighted regression was performed to evaluate the relation between PM_2.5_ concentrations and lung cancer mortality for males, females and for both sexes combined, in 2008, based on newly available long-term data. Lung cancer fatalities from long-term exposure to PM_2.5_ were calculated according to studies by Pope III *et al* and the WHO air quality guidelines (AQGs).

**Setting:**

31 provinces in China.

**Results:**

PM_2.5_ was associated with the lung cancer mortality of males, females and both sexes combined, in China, although there were exceptions in several regions, for males and females. The number of lung cancer fatalities calculated by the WHO AQGs ranged from 531 036 to 532 004, whereas the number calculated by the American Cancer Society (ACS) reached 614 860 after long-term (approximately 3–4 years) exposure to PM_2.5_ concentrations since 2008.

**Conclusions:**

There is a positive correlation between PM_2.5_ and lung cancer mortality rate, and the relationship between them varies across the entire country of China. The number of lung cancer fatalities estimated by ACS was closer to the actual data than those of the WHO AQGs. Therefore, the ACS estimate of increased risk of lung cancer mortality from long-term exposure to PM_2.5_ might be more applicable for evaluating lung cancer fatalities in China than the WHO estimate.

**Strengths and limitations of this study**

- This study provides an important contribution to the limited literature available on the association between Particulate Matter (PM)_2.5_ concentrations and mortality related to lung cancer in China, based on the newly published Annual Average PM_2.5_ gridded data and gridded population data.
- This study is the first in which geographically weighted regression models were applied to explore the spatial variation of the relation between PM_2.5_ concentrations and lung cancer mortality rates in China.
- The data on lung cancer mortality rates used in this study were estimates, which may induce bias when evaluating the association between PM_2.5_ concentrations and lung cancer mortality.

## Introduction

China has experienced serious air pollution in recent years. Public concern in Beijing boiled over in early 2013, when degradation of the air quality over several days reached some of the highest levels in recent years.^1^ Similar events occurred in many other cities, for example, Tianjin, Wuhan, Chongqing, etc. Particularly in winter, most of northern China is shrouded in thick fog and haze for extended periods of time. Impassioned debates about PM (Particulate Matter) air pollution and its corresponding health effects are ongoing. Epidemiological studies have consistently shown an association between particulate air pollution and various adverse health impacts. Several landmark review studies have indicated that exposure to fine particles (ie, PM_2.5_, particles with an aerodynamic diameter less than 2.5 µm) was associated with premature death and increased morbidity from respiratory and cardiovascular disease.^2–4^ Accurate ambient PM_2.5_ concentration measurements are critical for epidemiological studies on chronic human exposure but remain a challenge for large geographical areas.^5^ Most of the exposure–response associations have been derived from data gathered in the USA and a few European countries in which PM_2.5_ is routinely monitored.^6^ For example, existing studies have found consistent relations between exposure to fine particles and premature death across the Harvard Six-Cities area and other locations in the USA.^3^ Using ambient monitoring data in conjunction with the photochemical model, Fann and Risley^7^ estimated that 130 000 PM_2.5_-related deaths resulted from degraded air quality levels in the USA during 2005. Epidemiological studies have reported that PM_2.5_ has a strong association with medical conditions such as asthma, cardiovascular problems, respiratory infections and lung cancer.^8^ ^9^ There have also been many previous studies reporting a positive relationship between PM_2.5_ and lung cancer, and long-term exposure to high PM_2.5_ concentrations can increase the mortality rate from lung cancer.^10–13^ A well-known study based on prospective cohort data collected by the American Cancer Society (ACS)^14^ reported that long-term exposure to PM_2.5_ concentrations had significant effects on survival, and each 10 µg/m^3^ increase was associated with an increase in lung cancer mortality risk of approximately 8%. In 2005, the WHO published their air quality guidelines (AQGs) and concluded that health risks could increase with long-term exposure (ie, 3–4 years) to PM_2.5_ concentrations over 10 µg/m^3^. In addition, WHO established three interim targets (IT) for PM_2.5_.^15^ During recent years, in China, particularly in some large cities, an increase in mortality from lung cancer was observed at the same time that the number of smokers decreased and medical conditions improved, and studies on the link between lung cancer and particulate pollution are receiving increasing attention as the air quality deteriorates. Moreover, Hu and Jiang^16^ reported that long-term exposure to fine particulate air pollution was an important risk factor for lung cancer in China based on studies in the USA and European countries. Therefore, it is necessary to investigate the association between PM_2.5_ and mortality from lung cancer and to estimate the potential risks of mortality from lung cancer caused by exposure to PM_2.5_ in China for epidemiological studies.

However, the lack of exposure estimates has been a serious limiting factor for evaluating the short-term and long-term health outcomes of fine-particle pollution in China.^6^ ^16^ Particulate matter with an aerodynamic diameter of less than 10 µm (PM_10_) has been measured by the State Environmental Protection Administration of China (SEPAC) in only 111 key cities since 2004. Studies on the health effects associated with particulate matter were conducted in those cities based on PM_10_ data,^17^ ^18^ but PM_2.5_ concentrations were not reported until 2012. Beijing is an example of a city where the PM_2.5_ observations have only been available since late 2012. Fortunately, in June 2013, the Global Annual PM_2.5_ Grids, with a spatial resolution of 0.5°×0.5° for 2001–2010, were published by the Battelle Memorial Institute and the Center for International Earth Science Information Network (CIESIN)/Columbia University.^19^ This dataset provides a continuous surface of concentrations in micrograms per cubic metre of PM_2.5_ for use in health and environmental research.

The first objective of this paper is to explore the association between PM_2.5_ and mortality from lung cancer using a Geographically weighted regression (GWR) model based on the long-term and newly refined data; the second objective is to estimate the potential risks of mortality associated with lung cancer caused by exposure to high PM_2.5_ concentrations for comparison with the ACS study and to evaluate performance relative to the WHO AQGs.

## Methods

### Data acquisition

**Figure 1:**
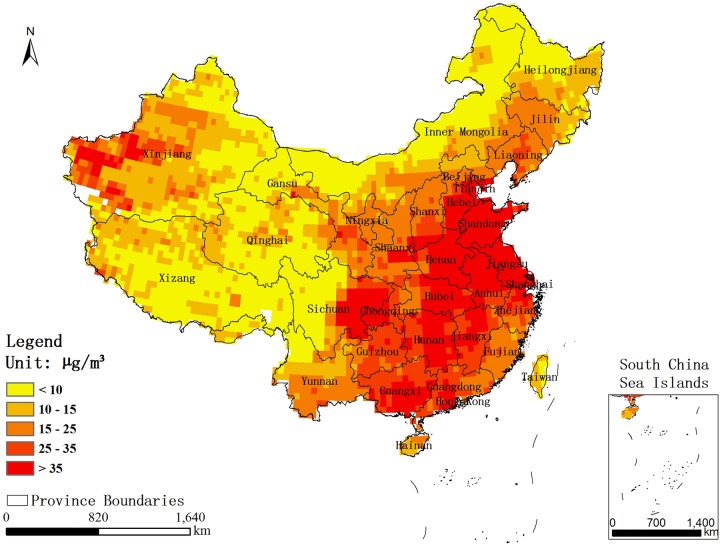
The estimated distribution of PM_2.5_ concentrations in 2008. PM, Particulate Matter.

The global annual average PM_2.5_ gridded data sets for the period 2001–2010^19^ were obtained from the website of the Socioeconomic Data and Applications Center (SEDAC); the spatial resolution of the data sets was 0.5°×0.5°. The data sets were generated by two researchers at Columbia University, based on the work of van Donkelaar *et al*.^20^ The conversion factor, which accounts for the spatio–temporal relationship between PM_2.5_ and aerosol optical depth (AOD), developed by van Donkelaar, was treated as a constant from 2001 to 2010 after minor processing; then, the monthly mean conversion factors were multiplied by the satellite AOD to calculate the PM_2.5_ concentration of each grid cell. Finally, the annual average surface PM_2.5_ concentrations were obtained by averaging the monthly estimates over each year.^19^ The PM_2.5_ gridded data from 2008 in China, used in this paper, were processed using ArcGIS 10.1, for better match with other data sets. Figure 1 shows the estimated distribution of PM_2.5_ concentrations in 2008.

**Figure 2:**
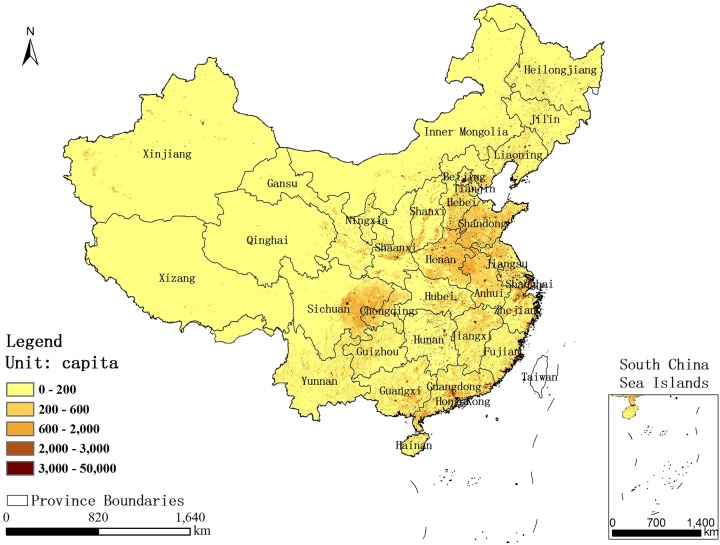
Spatial distribution of population in China based on SNPC. SNPC, Sixth National Population Census.

The gridded population data for China, used in this study, were based on the Sixth National Population Census (SNPC).^21^ To take advantage of the spatially explicit PM_2.5_ concentration data, we transformed the census data into spatial grids based on the administrative districts, which is an effective method for calculating the spatial distribution of a population with land cover data on the scale of 1:10 million. A multivariate model was developed to calculate the various population factors on different land cover types.^22^ The spatial population grid data were subsequently obtained with the support of the Geographic Information System (GIS) at a resolution of 1 km^2^. The result was modified by the DEM (Digital Elevation Model) data, and the resident point data, and was validated by the census data from several randomly selected towns.^22^ Finally, we analysed the status of the population distribution exposed to different PM_2.5_ concentrations, and estimated the lung cancer fatalities using risk estimates from the ACS study and WHO AQGs. Figure 2 shows the gridded population data in China, based on SNPC with a resolution of 1 km^2^.

**Figure 3:**
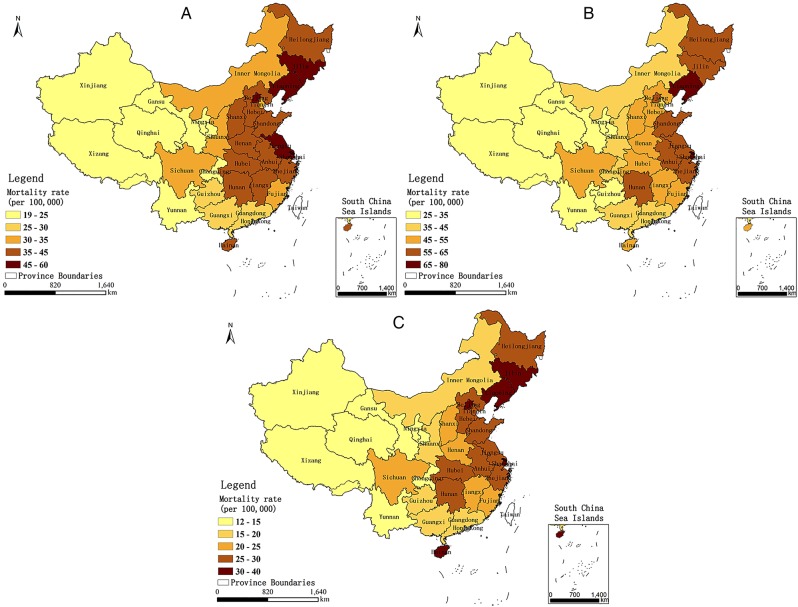
Distribution of mortality from lung cancer of (A) both sexes, and (B) males and (C) females, in China, by province, during 2008.

The data on mortality from lung cancer used in this study were taken from a population-based cross-sectional study.^23^ Population-based cancer registries are not well established and the epidemiological data for cancer in China have, so far, been limited. Based on population coverage and the accuracy of the available mortality estimates for the provinces, Li *et al* determined the lung cancer mortality rate for 31 provincial regions in China during 2008, using three estimation models. The model was fit and compared with prior data from the literature and was shown to successfully reflect the number of deaths caused by lung cancer in China.^23^ Owing to its reliability for mortality from lung cancer by province, these data can be considered as a valuable scientific reference for epidemiology until a new and more accurate lung cancer mortality report is published in China. Figure 3A–C shows the distribution of mortality from lung cancer in China by province during 2008, for both sexes combined, and for males and females separately.

### Statistics analysis

For this ecological study, a GWR was used to estimate the relation between PM_2.5_ concentrations and mortality from lung cancer for both sexes combined, and for males and females separately, in 2008. Use of GWR for environmental studies has been extensively described in recent publications^24^ ^25^; hence, only a brief summary is provided here. Using the ordinary least squares (OLS) method, the GWR was extended to explore spatial heterogeneity by building a local regression model.^26^ In the GWR model, the spatial locations of the data points are embedded in the regression parameters, and local estimates of the parameters are made. The GWR can indicate the influence of independent variables on dependent variables with a change in spatial location, demonstrating the spatial heterogeneity of the relation between an independent variable and dependent variable.^27^ Due to these special characteristics, the GWR model was used to explore spatial variance in the relation between PM_2.5_ concentrations and mortality from lung cancer for both sexes combined and separately for males and females, in this paper. Bandwidth, an important parameter for GWR, controls the degree of smoothing; the preferred bandwidth is determined by the interaction between the bandwidth and the complexity of the model, and there are usually two automatic methods for finding the bandwidth: CV, which minimises a cross-validation (CV) function, and AIC, which minimises the Akaike information criterion (AIC).^28^ In this study, an adaptive kernel of which the bandwidth was obtained by minimising the corrected Akaike information criterion (AICc) was chosen by evaluating the spatial configuration of the features.^29^

As mentioned above, an annual average PM_2.5_ concentration of 10 µg/m^3^ was regarded by WHO as the long-term guideline value for PM_2.5_; long-term exposure to an annual average concentration of more than 10 µg/m^3^ could increase mortality from lung cancer.^15^ A study based on the ACS CPS-II cohort concluded that, for each 10 µg/m^3^ increase in long-term exposure to PM_2.5_ concentrations, there was an associated increase in the risk of lung cancer mortality of approximately 8%.^14^ In order to analyse the population status within the scope of different PM_2.5_ concentrations, we classified the PM_2.5_ concentrations into six levels artificially (level 1 (0–10 µg/m^3^), level 2 (10–20 µg/m^3^), level 3 (20–30 µg/m^3^), level 4 (30–40 µg/m^3^), level 5 (40–50 µg/m^3^) and level 6 (50–60 µg/m^3^)), taking 10 µg/m^3^ change in PM_2.5_ as an increment. We assumed that there was no risk at concentration of 0 µg/m^3^, thus, an increase from 0 to 10 µg/m^3^ would yield an 8% increase in risk according to the study by Pope III *et al*. So when we estimated the number of lung cancer fatalities in level 1, we took the mean value of the two increased risks as the increase rate in the risk of lung cancer mortality, which was 4%. Similarly, an increase from 10 to 20 µg/m^3^ would also yield an 8% increase in risk, and the increase in the risk of lung cancer mortality in level 2 would be 12%; and the increase in the risk of lung cancer mortality in level 3, level 4, level 5 and level 6 were thereby 20%, 28%, 36% and 44%, respectively. The WHO defined 10 µg/m^3^ as the long-term guideline value for PM_2.5_ together with three ITs of 35 µg/m^3^ (IT-1), 25 µg/m^3^ (IT-2) and 15 µg/m^3^ (IT-3); long-term exposure to IT-1, IT-2 and IT-3 levels increased the mortality risk by approximately 15%, 9% and 3%, respectively.^15^ Tables 1 and 2 show the increased risk of mortality from lung cancer as a result of long-term exposure to an annual average PM_2.5_ concentration of more than 10 µg/m^3^ reported in the studies by Pope III *et al* and WHO, respectively.

**Table 1:** The increased risk (R) of mortality from lung cancer resulting from long-term exposure to the annual average PM_2.5_ concentrations, as reported in the study by Pope III *et al*

Levels*	PM_2.5_ concentrations (µg/m^3^)	R (%)
Level 6	50–60	44
Level 5	40–50	36
Level 4	30–40	28
Level 3	20–30	20
Level 2	10–20	12
Level 1	0–10	4

*Levels in this table refer to the PM_2.5_ concentration intervals that humans have long-term exposure to.

PM, Particulate Matter.

**Table 2:** The increased risk (R) of mortality from lung cancer resulting from long-term exposure to the annual average PM_2.5_ concentrations, as reported by WHO AQGs

PM_2.5_ concentrations (µg/m^3^)	R (%)*
35–60	15
25–35	9
15–25	3
10–15	0–3

*10 µg/m^3^ was regarded as the long-term guideline value for PM_2.5_; thus, in this study, we assumed that the risk of lung cancer did not increase with long-term exposure to PM_2.5_ concentrations of less than 10 µg/m^3^.

AQGs, air quality guidelines; PM, Particulate Matter.

Based on these data, the potential risk of mortality from lung cancer associated with exposure to high PM_2.5_ concentrations was evaluated in the following manner:

1. Step 1: Classify the PM_2.5_ concentrations in China reported by ACS and WHO AQGs.
2. Step 2: Analyse the population status in the zone of each PM_2.5_ concentration level using the gridded population dataset of China.
3. Step 3: Calculate the number of lung cancer fatalities at each level by multiplying the population size by the mortality rate from lung cancer and adding the increased lung cancer fatalities caused by PM_2.5_ air pollution as follows:1
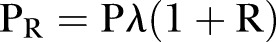

where λ is the mortality rate from lung cancer (both sexes) in China by province, P is the population size in the zone of each PM_2.5_ concentration level, and R is the increased risk of mortality from lung cancer resulting from long-term exposure to the annual average PM_2.5_ concentrations reported by the ACS and the WHO AQGs (see tables 1 and 2).

## Results

### Cumulative distribution of annual PM_2.5_ concentrations and the affected population in China

**Figure 4:**
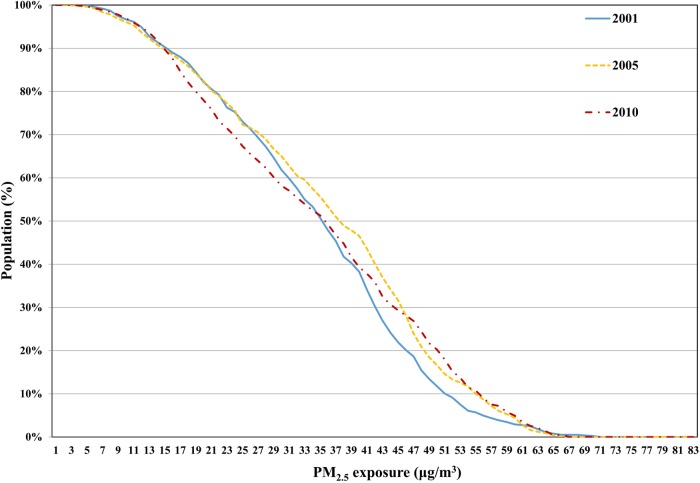
Cumulative distribution of PM_2.5_ and the affected population. PM, Particulate Matter.

Figure 4 shows the proportion of the Chinese population affected by the cumulative distribution of annual PM_2.5_ for 2001, 2005 and 2010; these data were obtained by overlaying the annual PM_2.5_ grids with the gridded population data. The proportion of the population exposed to PM_2.5_ concentrations of 15 µg/m^3^ increased from 2001 to 2005 and subsequently decreased (by approximately 50%) to levels observed in 2001 by 2010. The proportion of the population that was exposed to PM_2.5_ concentrations greater than 15 µg/m^3^ was approximately 90%, and this value changed little over the 10-year period. Thus, it is important for China to estimate the potential risk of mortality from lung cancer related to exposure to PM_2.5_, and to explore the relation between PM_2.5_ and mortality from lung cancer.

### Correlation between PM_2.5_ and mortality from lung cancer

Before the GWR could be conducted, a linear regression was performed to determine the initial relation between PM_2.5_ and the lung cancer mortality rate for males and females, as well as both sexes, in 2008. The results indicated that there were positive correlations between PM_2.5_ and lung cancer mortality rates for males, females and both sexes combined, with R values of 0.464, 0.475 and 0.471, respectively. In addition, an overall trend was observed such that provinces with higher PM_2.5_ concentrations had a greater mortality from lung cancer, but there were also exceptions to this trend (see figures 1 and 4). Thus, we constructed the GWR models to explore the spatial variation in the relation between PM_2.5_ and lung cancer mortality rate for both sexes, as well as for males and females.

**Figure 5:**
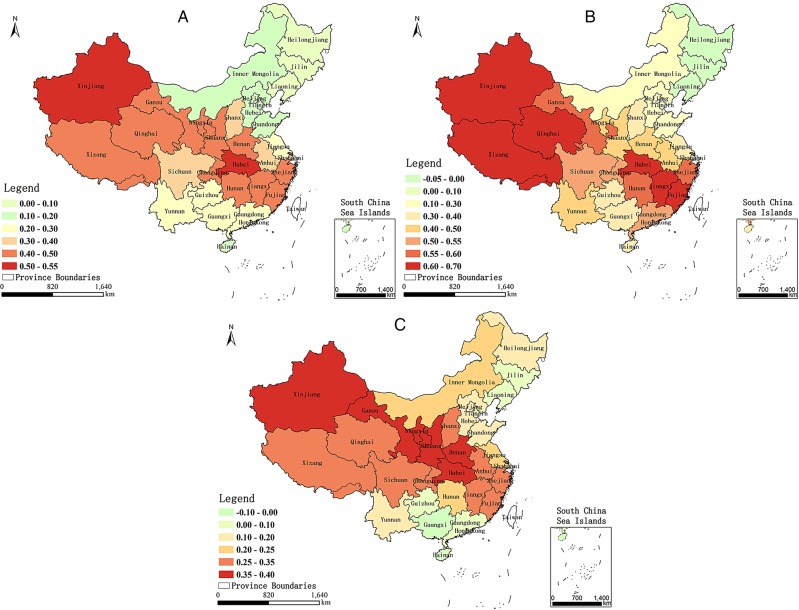
Maps of local coefficients of PM_2.5_ from the models for (A) both sexes, (B) males and (C) females. PM, Particulate Matter.

The adjusted R^2^ of the GWR models for males, females and both sexes combined was 0.654, 0.710 and 0.672, respectively, which indicates that the GWR models were a good fit for the data. Figure 5 shows the spatial variation in the relation between PM_2.5_ concentrations and the mortality from lung cancer for both sexes, and for males and females. To examine the spatial instability of the regression coefficients, the f-statistic, 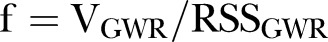 (where 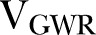 is the variance of the coefficients, and 
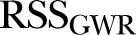
 is the residual sum of squares) was calculated.^30^ In this study, the ratios between the f-statistic and the degrees of freedom for each model were all larger than the critical value at the level of 0.05, which indicated that the spatial variation of the regression coefficients was not stationary.

Figure 5A shows the local coefficients of PM_2.5_ from the model for both sexes. In general, the local coefficients indicated that the influence of PM_2.5_ on the lung cancer mortality rate varies considerably across the country, and the entire country shows a positive correlation between mortality from lung cancer and PM_2.5_. This finding is consistent with the result obtained from the linear regression. Strong correlations with coefficients above 0.4 primarily appeared in the provinces of Xinjiang, Hubei, Jiangxi, Qinghai, Gansu, Xizang, Fujian, Zhejiang, Ningxia, Shaanxi, Chongqing, Hunan and Henan. Figure 5B shows the local coefficients of PM_2.5_ from the model for males. In general, the influence of the PM_2.5_ on the lung cancer mortality rate of males varied across the country. There were 28 provinces in China with a positive correlation between PM_2.5_ and lung cancer mortality rate in males, with strong correlations and coefficients above 0.4 primarily in the provinces of Xinjiang, Jiangxi, Qinghai, Xizang, Fujian, Hubei, Hunan, Gansu, Zhejiang, Chongqing, Sichuan, Guangdong, Shaanxi, Ningxia, Anhui, Henan and Yunnan. However, the sign of the local coefficient changed in the three Northeastern provinces (Heilongjiang, Liaoning and Jilin Provinces), which are the primary base of China’s heavy industries. Smoking and occupational exposure may be the primary contributors to mortality from lung cancer, particularly for men, in those regions.^31^ Figure 5C shows the local coefficients of PM_2.5_ from the model for females. As with males, the relation between PM_2.5_ and the lung cancer mortality rate of females varied spatially, and in 29 provinces within China, there was a positive correlation between the variables. Strong correlations were observed primarily in Ningxia, Shaanxi, Hubei, Gansu, Xinjiang, Henan, Shanxi, Zhejiang, Jiangxi, Chongqing, Anhui and Qinghai Provinces. Also similar to males, a negative correlation between PM_2.5_ and mortality from lung cancer was observed in Guangxi and Hainan Provinces, where a proportion of lung cancer in women may be attributable to cooking food.^32^ The overall trend of results from the GWR models was that increased PM_2.5_ concentrations are associated with increased mortality from lung cancer in China, although there are exceptions in several regions. The relation between PM_2.5_ and lung cancer mortality rates varied spatially, which indicates that PM_2.5_ is not likely to be the sole aetiological agent responsible for increased mortality from lung cancer in some regions and that each area has its own leading cause of lung cancer.

### Estimates of the potential risk of mortality from lung cancer associated with PM_2.5_

Table 3 shows the population affected by PM_2.5_ according to the study by Pope III *et al* and the WHO AQGs.

**Table 3:** Population affected by PM_2.5_ calculated according to the study by Pope III *et al* and WHO AQGs

	Estimation by Pope III *et al*	Estimation by WHO AQGs
Lung cancer deaths	614 860	531 036–532 004

AQGs, air quality guidelines; PM, Particulate Matter.

The number of lung cancer fatalities calculated by the WHO AQGs ranges from 531 036 to 532 004, whereas the number calculated by Pope III *et al* reached 614 860 after long-term (approximately 3–4 years) exposure to PM_2.5_ concentrations (table 3); the estimates of Pope III *et al* are larger than those of the WHO AQGs. Based on the cancer country profiles 2014,^33^ the number of lung cancer fatalities in China was approximately 595 000 in 2012, which is very close to the lung cancer fatalities based on excess risk estimates from Pope *et al*.

## Discussion

Many studies have been conducted to examine the relation between PM_2.5_ and mortality from lung cancer in the USA and European countries.^10^ ^12^ ^14^ ^34^ However, a lack of comprehensive data is the primary obstacle to investigating the association between PM_2.5_ and lung cancer mortality rates in China.^16^

This study is an important contribution to the limited literature available on the association between PM_2.5_ concentrations and lung cancer mortality rates in China; using spatially explicit PM_2.5_ and population data, our results provide strong evidence that long-term exposure to PM_2.5_ is a significant risk factor for lung cancer. In addition, this study also demonstrates spatial variation in the relation between PM_2.5_ and the lung cancer mortality rates for males, females and for both sexes combined, using GWR models; these models indicate that the influence of PM_2.5_ on lung cancer mortality rates varies with geographic location and that in some areas, aetiological agents other than PM_2.5_ might influence the lung cancer mortality rates. However, to account for the many factors that may affect lung cancer mortality rates and their complexities, additional statistical and experimental data are required. Based on the studies of Pope III *et al* and the WHO AQGs, this ecological study also provided an estimation of lung cancer fatalities following long-term (approximately 3–4 years) exposure to PM_2.5_ concentrations. The results indicated that the estimates of Pope III *et al* were more accurate than those calculated by the WHO AQGs, and the increased risk of lung cancer mortality as a result of long-term exposure to the annual average PM_2.5_ concentrations reported by Pope III *et al* might be more applicable for evaluating lung cancer fatalities in China.

## Conclusions

The goal of this study was to explore the association between PM_2.5_ concentrations and lung cancer mortality rates, and also to estimate the increased risk of lung cancer mortality caused by long-term exposure to PM_2.5_ in China, based on spatially explicit PM_2.5_ and population data. We draw the following conclusions:

Long-term exposure to PM_2.5_ is an important risk factor for lung cancer, and there are positive correlations between PM_2.5_ and lung cancer mortality rate for both sexes, and for males and females, in China.

The relation between PM_2.5_ concentrations and lung cancer mortality rates varies spatially, and, in addition to PM_2.5_, there are other aetiologic agents that may influence the lung cancer mortality rate in different areas of China.

In comparison with the WHO AQGs, the lung cancer fatalities estimated by the ACS are more accurate, and the ACS estimates of increased risk of lung cancer mortality rates from long-term exposure to the annual average PM_2.5_ concentrations could be used for evaluating lung cancer fatalities in China.

This paper provides the first overall estimation of lung cancer mortality in China at a national scale and might serve as a valuable guideline for ecological studies. However, the problem is complex, and this study contains uncertainties owing to its ecological nature; the latency period associated with lung cancer cannot be considered due to the deficient PM_2.5_ data. Thus, systematic studies are needed in the future.
